# Opioid Prescribing After Surgery in the United States, Canada, and Sweden

**DOI:** 10.1001/jamanetworkopen.2019.10734

**Published:** 2019-09-04

**Authors:** Karim S. Ladha, Mark D. Neuman, Gabriella Broms, Jennifer Bethell, Brian T. Bateman, Duminda N. Wijeysundera, Max Bell, Linn Hallqvist, Tobias Svensson, Craig W. Newcomb, Colleen M. Brensinger, Lakisha J. Gaskins, Hannah Wunsch

**Affiliations:** 1 Department of Anesthesia, St. Michael’s Hospital, Toronto, Ontario, Canada; 2 Department of Anesthesia, University of Toronto, Toronto, Ontario, Canada; 3 Li Ka Shing Knowledge Institute, St. Michael’s Hospital, Toronto, Ontario, Canada; 4 Leonard Davis Institute of Health Economics, University of Pennsylvania, Philadelphia; 5 Department of Anesthesiology and Critical Care, University of Pennsylvania Perelman School of Medicine, Philadelphia; 6 Penn Center for Perioperative Outcomes Research and Transformation (CPORT), University of Pennsylvania Perelman School of Medicine, Philadelphia; 7 Division of Epidemiology and Centre for Pharmacoepidemiology, Karolinska Institutet, Solna, Sweden; 8 Department of Internal Medicine, Danderyd University Hospital, Danderyd, Sweden; 9 ICES Central, Toronto, Ontario, Canada; 10 Sunnybrook Research Institute, Sunnybrook Health Sciences Center, Toronto, Ontario, Canada; 11 Department of Anesthesiology, Perioperative, and Pain Medicine, Brigham and Women’s Hospital, Harvard Medical School, Boston, Massachusetts; 12 Division of Pharmacoepidemiology and Pharmacoeconomics, Department of Medicine, Brigham and Women’s Hospital, Harvard Medical School, Boston, Massachusetts; 13 Section for Anaesthesiology and Intensive Care Medicine, Department of Physiology and Pharmacology, Karolinska Institutet, Solna, Sweden; 14 Perioperative Medicine and Intensive Care, Karolinska University Hospital, Solna, Sweden; 15 Clinical Epidemiology Division, Karolinska Institutet, Solna, Sweden; 16 Center for Clinical Epidemiology and Biostatistics, Department of Biostatistics, Epidemiology and Informatics, Perelman School of Medicine, University of Pennsylvania, Philadelphia; 17 Department of Critical Care Medicine, Sunnybrook Health Sciences Centre, Toronto, Ontario, Canada; 18 Interdepartmental Division of Critical Care Medicine, University of Toronto, Toronto, Ontario, Canada

## Abstract

**Importance:**

Small studies and anecdotal evidence suggest marked differences in the use of opioids after surgery internationally; however, this has not been evaluated systematically across populations receiving similar procedures in different countries.

**Objective:**

To determine whether there are differences in the frequency, amount, and type of opioids dispensed after surgery among the United States, Canada, and Sweden.

**Design, Setting, and Participants:**

This cohort study included patients without previous opioid prescriptions aged 16 to 64 years who underwent 4 low-risk surgical procedures (ie, laparoscopic cholecystectomy, laparoscopic appendectomy, arthroscopic knee meniscectomy, and breast excision) between January 2013 and December 2015 in the United States, between July 2013 and March 2016 in Canada, and between January 2013 and December 2014 in Sweden. Data analysis was conducted in all 3 countries from July 2018 to October 2018.

**Main Outcomes and Measures:**

The main outcome was postoperative opioid prescriptions filled within 7 days after discharge; the percentage of patients who filled a prescription, the total morphine milligram equivalent (MME) dose, and type of opioid dispensed were compared.

**Results:**

The study sample consisted of 129 379 patients in the United States, 84 653 in Canada, and 9802 in Sweden. Overall, 52 427 patients (40.5%) in the United States were men, with a mean (SD) age of 45.1 (12.7) years; in Canada, 25 074 patients (29.6%) were men, with a mean (SD) age of 43.5 (13.0) years; and in Sweden, 3314 (33.8%) were men, with a mean (SD) age of 42.5 (13.0). The proportion of patients in Sweden who filled an opioid prescription within the first 7 days after discharge for any procedure was lower than patients treated in the United States and Canada (Sweden, 1086 [11.1%]; United States, 98 594 [76.2%]; Canada, 66 544 [78.6%]; *P* < .001). For patients who filled a prescription, the mean (SD) MME dispensed within 7 days of discharge was highest in United States (247 [145] MME vs 169 [93] MME in Canada and 197 [191] MME in Sweden). Codeine and tramadol were more commonly dispensed in Canada (codeine, 26 136 patients [39.3%]; tramadol, 12 285 patients [18.5%]) and Sweden (codeine, 170 patients [15.7%]; tramadol, 315 patients [29.0%]) than in the United States (codeine, 3210 patients [3.3%]; tramadol, 3425 patients [3.5%]).

**Conclusions and Relevance:**

The findings indicate that the United States and Canada have a 7-fold higher rate of opioid prescriptions filled in the immediate postoperative period compared with Sweden. Of the 3 countries examined, the mean dose of opioids for most surgical procedures was highest in the United States.

## Key Points

**Question:**

Do rates of opioid prescriptions dispensed after surgical procedures differ among countries?

**Findings:**

In this cohort study, more than 70% of surgical patients in the United States and Canada filled opioid prescriptions after 4 surgical procedures compared with only 11% in Sweden. Of the 3 countries examined, the United States had the highest average dose of opioid prescriptions for most surgical procedures.

**Meaning:**

There is very large variability in the use of opioids after surgery in different countries, suggesting the potential to reevaluate prescribing practices.

## Teaser

This cohort study determines whether there are differences in the frequency, amount, and type of opioids dispensed after surgery among the United States, Canada, and Sweden.

## Introduction

Opioids are routinely prescribed for postoperative pain management in many countries. Thus, surgery is among the most common indications for opioid initiation.^1,2^ Data from the United States suggest that there has been an increase in the amount of opioids dispensed following minor surgical procedures^3^ and that many US patients receive more opioids than necessary to treat their short-term pain.^4^ Excessive postoperative opioid prescribing has been associated with increased risks of drug diversion, new long-term opioid use, and the development of opioid use disorder.^4,5,6^

While rates of opioid consumption are known to vary across countries,^7,8,9^ few systematic data exist to characterize international differences in opioid use for specific indications, such as pain relief after surgery.^10^ These comparisons can inform hypotheses regarding the cultural or structural drivers of overprescribing and highlight alternative approaches to inform targets for improvement. We compared opioid prescriptions dispensed during the first 7 and 30 days after 4 low-risk surgical procedures in the United States, Canada, and Sweden. We hypothesized that there would be significant differences among the 3 countries regarding the frequency, amount, and type of opioids dispensed after surgery.

## Methods

### Study Setting

This was a retrospective cohort study of patients undergoing surgery in the United States, Canada, and Sweden. The United States and Canada were selected because they have the highest per capita opioid consumption in the world,^8^ and Sweden was included as a European comparator that also had population-level databases with detailed opioid prescription information available. The study was exempted from review by the institutional review boards at the University of Pennsylvania (Philadelphia, Pennsylvania, United States) and Sunnybrook Health Sciences (Toronto, Ontario, Canada) and approved by the Regional Ethical Review Board in Stockholm, Sweden. Informed consent was not required because data were deidentified. Analysis of the data occurred between July 2018 and October 2018. This study followed the Strengthening the Reporting of Observational Studies in Epidemiology (STROBE) reporting guideline for cohort studies.^11^

### Data Sources

Data were available from January 1, 2013, through December 31, 2015, in the United States; July 1, 2013, through March 31, 2016, in Canada; and January 1, 2013, through December 31, 2014, in Sweden. These years were selected based on the availability of data with overlapping time periods across countries to maximize sample size while limiting the potential effect of temporal trends on prescribing practices. Data from the United States were abstracted from Clinformatics Data Mart (Optum), a database that includes claims from a nationwide commercial insurer for approximately 13.5 million beneficiaries yearly. In Canada, data were obtained from 3 databases in the province of Ontario, ie, the Discharge Abstract Database, Same Day Surgery Database, and Narcotics Monitoring System. The data sets were linked using unique encoded identifiers and analyzed at ICES, a nonprofit research institute. These administrative data cover residents of Ontario who are eligible for universal health coverage, which represents approximately 13 million individuals and 40% of the Canadian population. Swedish data were collected from hospitals using the Orbit surgical planning system software (EVRY), which covers approximately 40% of the Swedish population. To acquire information on discharge dates, covariates, and drug exposure, surgical records were linked to the national patient register and the national prescribed drug register using the personal identification number assigned to all residents at birth or immigration.

### Inclusion and Exclusion Criteria

**Figure 1.**
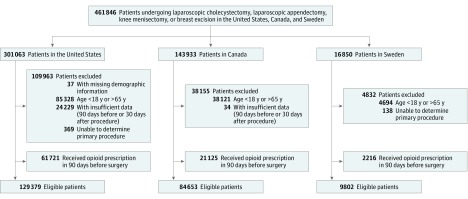
Flowchart of Cohort Formation in Each Country

We included all patients aged 18 to 64 years undergoing any one of the following 4 surgical procedures: (1) laparoscopic cholecystectomy, (2) laparoscopic appendectomy, (3) knee arthroscopy with meniscectomy, and (4) partial breast excision. These surgical procedures were chosen because they are frequently performed, low-risk procedures with common administrative or billing codes that uniquely identify them across all 3 countries (eTable 1 in the Supplement). Both elective and urgent procedures were considered for analysis in this study. Patients 65 years or older were excluded given the inability to accurately capture dispensed medications in the US database owing to Medicare enrollment. For patients undergoing more than 1 of these procedures during the study period, only the first procedure was included for analysis. Patients who received multiple eligible procedures during a single hospital admission were included in the sample if 1 procedure could be identified as the primary operation performed during the care episode. Within the US cohort, we excluded individuals with less than 90 days of available claims data before the date of hospital admission to limit missing data. Additionally, we excluded individuals with less than 30 days of follow-up data after hospital discharge. We also excluded individuals with missing demographic data and patients who filled a prescription for any opioid or opioid agonist during the 90 days preceding admission for surgery to avoid confounding from preoperative opioid use (Figure 1).

### Outcomes

The primary outcome was filling 1 or more prescriptions for an opioid analgesic in the first 7 days after surgery (days 0-6), where day 0 was defined as either the hospital discharge date or the date of surgery, if there was no hospitalization. Eligible opioid analgesics were codeine, fentanyl, hydrocodone, hydromorphone, ketobemidone, levorphanol, meperidine, morphine, oxycodone, oxymorphone, pentazocine, tramadol, and tapentadol. We excluded opioids prescribed in combination with a decongestant (eg, pseudoephedrine) or an antihistamine (eg, diphenhydramine), which we considered to be prescribed for indications other than postoperative analgesia. Secondary outcomes were the following: (1) the amount of opioid dispensed in the first prescription (ie, the first prescription filled days 0-6, including multiple prescriptions if all filled on the same day), (2) the amount dispensed in total during the first 30 days (days 0-29) using morphine milligram equivalents (MMEs) (eTable 2 in the Supplement),^12^ (3) the first prescription’s type of opioid, (4) the postoperative day of dispensing, (5) use of combination opioid products (ie, an opioid formulation that also contained acetaminophen, ibuprofen, aspirin, or another nonsteroidal anti-inflammatory drug), and (6) the MME of the first prescription, stratified by type of opioid.

### Baseline Characteristics

Baseline characteristics included age at the date of surgery, sex, prior inpatient hospitalizations within 90 days before admission for surgery, and comorbidities (Charlson Comorbidity Index ascertained using data from the index surgical admission). We also determined whether the surgery was performed as an outpatient or inpatient procedure and the length of hospital stay.

### Statistical Analysis

Data could not be pooled because of data use agreements, so data from each country were analyzed separately. For outcomes, we used proportions (95% CIs), means (SDs), and medians (interquartile ranges), both overall and within strata defined by surgical procedure. Table cells with fewer than 10 patients were not reported to ensure patient privacy. Unadjusted comparisons were made between countries using χ^2^ tests for categorical variables and analysis of variance for continuous variables using the summary data. To assess whether differences in the likelihood of filling an opioid prescription across countries may have been explained by variations in hospital length of stay after surgery, we repeated all analyses in a subgroup restricted to patients who underwent outpatient procedures. All available data were used, and no prespecified sample size calculation was performed. Statistical tests were 2-tailed, with significance defined as *P* < .05. Analyses were performed in R version 3.4.1 (R Foundation) and SAS version 9.4 (SAS Institute).

## Results

### Cohort Characteristics

The final study sample consisted of 129 379 patients in the United States, 84 653 in Canada, and 9802 in Sweden. Overall, 52 427 patients (40.5%) in the United States were men, with a mean (SD) age of 45.1 (12.7) years; in Canada, 25 074 patients (29.6%) were men, with a mean (SD) age of 43.5 (13.0) years; and in Sweden, 3314 (33.8%) were men, with a mean (SD) age of 42.5 (13.0) years. Among the 191 100 individuals in the United States who underwent 1 of 4 selected surgical procedures and had sufficient data, 61 721 patients (32.3%) were excluded because they filled an opioid prescription in the 90 days before admission, compared with 21 125 of 105 778 (20.0%) in Canada and 2216 of 12 018 (18.4%) in Sweden (Figure 1). The distribution of procedures varied between the countries (Table), with the United States having the highest proportion of patients undergoing knee meniscectomies (44 060 [34.1%] vs 6975 [8.2%] in Canada and 1791 [18.3%] in Sweden) and a smaller percentage undergoing a breast excision (16 170 [12.5%] vs 18 143 [21.4%] in Canada and 2247 [22.9%] in Sweden). Most baseline characteristics were similar across the 3 countries, including age, Charlson Comorbidity Index scores, and the proportion of patients admitted to the hospital at least 1 day in the 90 days preceding admission for surgery. For the index surgery, the United States had the most outpatients (111 883 [86.5%] vs 56 956 [67.3%] in Canada and 4742 [48.4%] in Sweden); Sweden had the largest percentage of patients hospitalized for 2 days or longer (2512 [25.6%] vs 13 276 [15.7%] in Canada and 12 754 [9.9%] in the United States). Patient characteristics stratified by surgical procedure are presented in eTable 3 in the Supplement.

**Table.** Baseline Characteristics of Patients in 3 Countries Who Underwent Laparoscopic Appendectomy, Laparoscopic Cholecystectomy, Knee Meniscectomy, or Breast Excision

Characteristic	No. (%)			*P* Value^a^
	United States (n = 129 379)	Canada (n = 84 653)	Sweden (n = 9802)	
Type of procedure				
Laparoscopic cholecystectomy	46 781 (36.2)	39 098 (46.2)	3530 (36.0)	<.001
Laparoscopic appendectomy	22 368 (17.3)	20 437 (24.1)	2234 (22.8)	
Knee meniscectomy	44 060 (34.1)	6975 (8.2)	1791 (18.3)	
Breast excision	16 170 (12.5)	18 143 (21.4)	2247 (22.9)	
Men	52 427 (40.5)	25 074 (29.6)	3314 (33.8)	<.001
Age, y				
Mean (SD)	45.1 (12.7)	43.5 (13.0)	42.5 (13.0)	NA
18-39	43 572 (33.7)	31 628 (37.4)	3895 (39.7)	<.001
40-54	51 206 (39.6)	32 471 (38.4)	3751 (38.3)	
55-64	34 601 (26.7)	20 554 (24.3)	2156 (22.0)	
Charlson Comorbidity Index score^b^				
0	108 568 (83.9)	73 314 (86.6)	8387 (85.6)	<.001
1	4932 (3.8)	2746 (3.2)	148 (1.5)	
≥2	15 879 (12.3)	8593 (10.2)	1267 (12.9)	
Overnight hospital stay in 90 d before admission	2537 (2.0)	3279 (3.9)	450 (4.6)	<.001
Length of hospital stay associated with surgery, d				
0	111 883 (86.5)	56 956 (67.3)	4742 (48.4)	<.001
1	4742 (3.7)	14 421 (17.0)	2548 (26.0)	
≥2	12 754 (9.9)	13 276 (15.7)	2512 (25.6)	

^a^ Calculated with χ^2^ test for categorical variables or analysis of variance for continuous variables.

^b^ Calculated based on data from the index surgical stay only.

### Filled Opioid Prescriptions After Discharge

**Figure 2.**
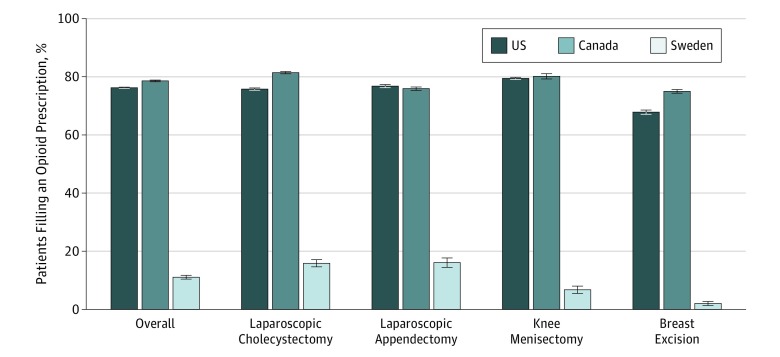
Percentage of Patients Filling an Opioid Prescription During First 7 Days After Surgery Error bars represent 95% CIs.

The overall proportion of patients who filled an opioid prescription within the first 7 days of discharge was similar between the United States and Canada at 98 594 (76.2%) and 66 544 (78.6%), respectively (Figure 2). This number was significantly lower in Sweden, where only 1086 patients (11.1%) filled an opioid prescription within the first 7 days after discharge (*P* < .001). This pattern was consistent across all 4 surgical procedures, with very similar numbers of patients filling prescriptions in the United States and Canada and much lower numbers in Sweden (Figure 2).

**Figure 3.**
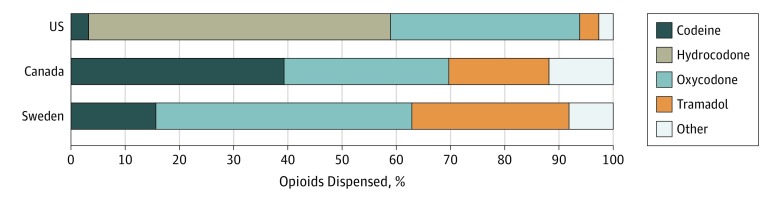
Distribution of Types of Opioids in First Prescriptions Filled

There were significant differences by country in the type of opioid prescription filled. Codeine was commonly prescribed in Canada (26 136 [39.3%]) and Sweden (170 [15.7%]) but rarely used in the United States (3210 [3.3%]). Similarly, tramadol was often prescribed in Sweden (315 [29.0%]) and Canada (12 285 [18.5%]) but represented only 3.5% of opioid prescriptions (3425 patients) in the United States (Figure 3; eTable 4 in the Supplement). The use of combination opioid formulations was higher in the United States and Canada (89 390 [90.7%] and 57 346 [86.2%], respectively) than in Sweden (171 [15.7%]).

### Dose of Opioid Dispensed

**Figure 4.**
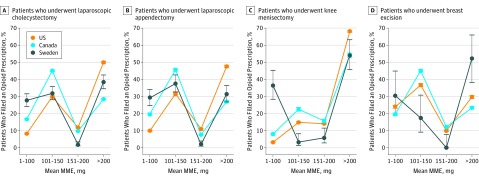
Distribution of Morphine Milligram Equivalents (MMEs) Dispensed for First Prescriptions Filled Error bars represent 95% CIs.

Among patients who filled an opioid prescription, the mean (SD) amount of opioid dispensed for the initial prescription was highest in the United States (247 [145] MME vs 169 [93] MME in Canada and 197 [191] MME in Sweden) (eTable 4A in the Supplement). Within each surgical procedure, the United States had the largest percentage of patients who filled a prescription for more than 200 MME total, except for breast excision, where 26 of 46 individuals in Sweden who underwent breast excision and received an opioid within 7 days filled a prescription with more than 220 MME (Figure 4). Stratified by type of opioid, the mean MME dispensed in the United States was higher regardless of the type of opioid prescribed (eTable 5 in the Supplement), suggesting that the overall differences observed in MMEs were not attributable to differences in the type of agent prescribed across countries. Among single opioid prescriptions in the United States across all surgical procedures, the mean (SD) MME dispensed ranged from 147 (68) MMEs for codeine up to 444 (269) MMEs for morphine.

### Opioid Prescriptions Within 30 Days of Discharge

Within 30 days of discharge, 58 867 individuals in the United States (45.5%) had received opioids in excess of 200 MME compared with 21 931 patients in Canada (25.9%) and 526 in Sweden (5.4%) (eTable 6 and the eFigure in the Supplement). Among those who filled at least 1 opioid prescription within 30 days, a greater percentage of patients filled 2 or more prescriptions in the United States (13 291 [13.1%]) and Sweden (157 [13.0%]) compared with Canada (4828 [7.2%]) (eTable 6 in the Supplement).

In a sensitivity analysis where only outpatient procedures were included, the percentage of patients who filled an opioid prescription within 7 days of surgery was similar to the primary analysis. Most notably, the number of patients filling an opioid prescription within 7 days of outpatient surgery in Sweden was 223 (4.7%) vs 86 085 (76.9%) in the United States and 45 953 (80.7%) in Canada (eTable 7 in the Supplement).

## Discussion

In this study of 223 834 opioid-naive patients undergoing 4 common surgical procedures across 3 developed countries, we observed differences in the frequency, amount, and type of opioids prescribed after surgery. Patients in Canada and the United States filled opioid prescriptions within the first 7 days after discharge at a rate nearly 7-fold higher than individuals in Sweden. Even though the frequency of prescriptions filled in Canada and the United States was similar, US patients received significantly higher quantities of opioids as measured by total MME dispensed compared with patients treated in Canada. While there were differences in the type of opioids prescribed, higher amounts were prescribed in the United States across all medication types. These findings were consistent for each of the 4 surgical procedures we assessed as well as in a subgroup analysis where only outpatient procedures were considered.

Extensive research in the United States suggests that overprescribing opioids for short-term pain may be widespread,^4,13^ and multiple US states have undertaken health policy interventions with the goal of reducing overprescribing of opioids as a means of reducing harm.^14^ In this context, the present comparisons highlight alternative approaches to treating postoperative pain that may be informative for clinicians and policy makers in countries with high rates of opioid prescribing after surgery, such as the United States and Canada. Notably, we were able to identify populations of opioid-naive patients in each of our 3 study countries that were similar overall in terms of age, demographic characteristics, and medical history who underwent 4 common and well-defined surgical procedures that use standard approaches and are likely to be associated with similar degrees of tissue trauma. As such, our results are unlikely to be attributable to differences across countries in patients’ individual medical characteristics, surgical procedure type, or prior use of opioids. Instead, our findings are more likely to point to differences in practitioners’ approaches to opioid prescribing,^15^ public attitudes regarding the role of opioids in treating pain,^16,17,18^ and broader structural factors related to drug marketing and regulation that may encourage or constrain postoperative opioid prescribing.^19,20^

We also noted large variation across countries in the types of opioids dispensed after surgery. Most notably, codeine and tramadol together accounted for approximately 58% and 45% of postoperative prescriptions in Canada and Sweden compared to only 7% in the United States. Tramadol and codeine rely on metabolites for their primary analgesic effect, leading to differing analgesic effects and risk profiles across individuals based on genetic polymorphisms.^21^ While prescribers may view these so-called weak opioids as safer alternatives, data suggests that both codeine and tramadol have the potential for misuse and life-threatening adverse effects.^22,23,24^ A possible explanation for these differences in prescribing is that, during the study period, low-dose codeine was available over the counter in Canada. Further, tramadol is still not considered a controlled or scheduled substance in Canada; however, it became a controlled substance in 2007 in Sweden^25^ and in 2014 in the United States.^26^ These findings suggest potential opportunities for reevaluation of analgesic selection practices in Canada and Sweden and educating prescribers regarding the variable effects with these medications.

### Limitations

This study has limitations. An important limitation is the absence of information on patients’ postoperative pain experiences; as such, we were unable to assess the overall quality of pain treatment across the countries examined. Indeed, it is possible that pain management may have been less effective for patients who did not receive opioids or were prescribed a lower amount. At the same time, previous evidence suggests that differences in opioid prescribing across developed countries are unlikely to be associated with differences in the quality of pain treatment patients receive. First, prior surveys conducted in all 3 countries studied here have demonstrated similar rates of patients experiencing moderate to severe pain and similar levels of satisfaction with postoperative analgesia.^27,28,29^ Second, opioids frequently remain unused after surgery in the United States, indicating potential prescribing out of proportion to need.^4,13^ Third, interventions in both Canada and the United States have successfully lowered postoperative opioid prescription rates without affecting the quality of pain treatment.^30,31^ Fourth, prior work has found a lack of correlation between the initial opioid prescription amount and the need for a refill prescription.^32^ This was also seen in our data, as the proportion of patients filling 2 or more prescriptions within 30 days of surgery was higher in the United States compared with Canada despite US patients receiving a greater amount of opioid as measured in terms of MMEs in the first 7 days after discharge.

There are also several additional limitations that should be considered when interpreting our results. First, the data collected represented prescriptions dispensed, and thus, we could not capture prescriptions written but not filled. Therefore, our data may not provide a complete understanding of physician prescribing behavior. Similarly, we were unable to determine how much opioid was actually consumed by patients and thus could not identify the proportion unused or diverted or whether patients obtained medication from other sources, such as directly from health care practitioners, family, or friends. Additionally, each national cohort consisted of a sample of patients that was not necessarily representative of the country’s entire population. For example, in the United States, only data on patients with commercial insurance were available, and in Canada, only patients treated in the province of Ontario were included. However, each county’s database encompassed a significant proportion of the overall population, and given the magnitude of variation observed, these differences were unlikely to explain our results. Further, we did not capture detailed perioperative data such as the use of regional anesthetic techniques. It is unclear how this would bias the results given that previous work suggests tremendous variation in the use of multimodal analgesia within a single country and that these techniques do not necessarily affect postoperative opioid consumption.^33,34^ This limitation also extended to the inability to account for postoperative complications in our analysis. However, the sensitivity analysis only included patients who underwent outpatient procedures and were thus unlikely to have experienced a complication.

## Conclusions

In summary, we observed differences in opioid prescribing after low-risk surgical procedures across 3 countries in North America and Europe. Patients treated in the United States and Canada received opioids after surgery more often and in higher doses compared with patients treated in Sweden. These findings highlight opportunities to encourage judicious use of opioids in the perioperative period in both the United States and Canada. Understanding the societal and cultural factors that influence these prescribing patterns could inform areas of further research and identify targets for future interventions.

## Notes

Supplement. **eTable 1.** Codes Used to Identify Surgical Procedures **eTable 2.** Postoperative Opioid Prescriptions **eTable 3.** Baseline Characteristics for Patients by Surgical Procedure **eTable 4.** First Postoperative Opioid Prescription Between Day 0 and Day 6 by Surgical Procedure **eTable 5.** Morphine Milligram Equivalents Prescribed in First Postoperative Opioid Prescription Between Day 0 and Day 6 by Surgical Procedure **eTable 6.** Opioid Prescriptions Filled Within 30 Days of Surgery or Date of Hospital Discharge by Surgical Procedure **eTable 7.** Sensitivity Analysis Excluding Patients Hospitalized for Surgery (Outpatient Surgery Only) **eFigure.** Distribution of Morphine Milligram Equivalents Dispensed for All Prescriptions Filled, Days 0 to 29, for Patients Who Filled an Opioid Prescription in Each Country, by Surgical Procedure
