# Supplementary material for: Opioid Prescribing After Surgery in the United States, Canada, and Sweden
Source: JAMA Netw Open. 2019 Sep 4;2(9):e1910734. doi: 10.1001/jamanetworkopen.2019.10734 (PMC6727684; doi:10.1001/jamanetworkopen.2019.10734)
Supplement: Supplement. — eTable 1. Codes Used to Identify Surgical Procedures eTable 2. Postoperative Opioid Prescriptions eTable 3. Baseline Characteristics for Patients by Surgical Procedure eTable 4. First Postoperative Opioid Prescription Between Day 0 and Day 6 by Surgical Procedure eTable 5. Morphine Milligram Equivalents Prescribed in First Postoperative Opioid Prescription Between Day 0 and Day 6 by Surgical Procedure eTable 6. Opioid Prescriptions Filled Within 30 Days of Surgery or Date of Hospital Discharge by Surgical Procedure eTable 7. Sensitivity Analysis Excluding Patients Hospitalized for Surgery (Outpatient Surgery Only) eFigure. Distribution of Morphine Milligram Equivalents Dispensed for All Prescriptions Filled, Days 0 to 29, for Patients Who Filled an Opioid Prescription in Each Country, by Surgical Procedure [file jamanetwopen-2-e1910734-s001.pdf]

Supplementary Online Content

Ladha KS, Neuman MD, Broms G, et al. Opioid prescribing after surgery in the United States, Canada, and Sweden. *JAMA Netw Open*. 2019;2(9):e1910734. doi:10.1001/jamanetworkopen.2019.10734

eTable 1. Codes Used to Identify Surgical Procedures
eTable 2. Postoperative Opioid Prescriptions
eTable 3. Baseline Characteristics for Patients by Surgical Procedure
eTable 4. First Postoperative Opioid Prescription Between Day 0 and Day 6 by Surgical Procedure
eTable 5. Morphine Milligram Equivalents Prescribed in First Postoperative Opioid Prescription Between Day 0 and Day 6 by Surgical Procedure
eTable 6. Opioid Prescriptions Filled Within 30 Days of Surgery or Date of Hospital Discharge by Surgical Procedure
eTable 7. Sensitivity Analysis Excluding Patients Hospitalized for Surgery (Outpatient Surgery Only)
eFigure. Distribution of Morphine Milligram Equivalents Dispensed for All Prescriptions Filled, Days 0 to 29, for Patients Who Filled an Opioid Prescription in Each Country, by Surgical Procedure

This supplementary material has been provided by the authors to give readers additional information about their work.

© 2019 Ladha KS et al. *JAMA Network Open.*

eTable 1. Codes Used to Identify Surgical Procedures

	Canadian Classification of Health Interventions Code	Current Procedural Terminology Codes	Nordic Meidco-Statistical Committee Classification of Surgical Procedures
Laparoscopic Cholecystectomy	1.OD.89DA<br>1.OD.89DT-AG<br>1.OD.89DT-AM<br>1.OD.89DT-AS<br>1.OD.89DT-BD<br>1.OD.89DT-GX<br>1.OD.89EC	47562, 47563, 47564	JKA21
Knee Arthroscopy - Meniscectomy	1.VK.87.DA<br>1.VK.89.DA	29881, 29880	NGD01, NGD11
Partial Excision of Breast	1.YM.87.^^	19301, 19302, 19120	HAB40, HAB99
Laparoscopic Appendectomy	1.NV.89.DA	44970	JEA01

© 2019 Ladha KS et al. *JAMA Network Open.*

eTable 2. Postoperative Opioid Prescriptions

Type of Opioid (Strength Units)*	Morphine Milligram Equivalent Conversion Factor
Codeine (mg)	0.15
Fentanyl tablets (mcg)	0.13
Hydrocodone (mg)	1
Ketobemidone	1
Levorphanol (mg)	11
Meperidine (mg)	0.1
Morphine (mg)	1
Oxycodone (mg)	1.5
Oxymorphone (mg)	3
Pentazocine (mg)	0.37
Tapentadol (mg)	0.4
Tramadol (mg)	0.1

*Only tablet forms of these medications were considered.

Source:

1) Centers for Medicare & Medicaid Services. Opioid Oral Morphine Milligram Equivalent (MME) Conversion Factors. https:\\www.cms.govMedicarePrescription-Drug-CoveragePrescriptionDrugCovContraDownloadsOpioid-Morphine-EQ-Conversion-Factors-vFeb-.pdf. Accessed: September 25, 2018.
2) Svendsen, K., Borchgrevink, P., Fredheim, O., Hamunen, K., Mellbye, A., & Dale, O. (2011). Choosing the unit of measurement counts: the use of oral morphine equivalents in studies of opioid consumption is a useful addition to defined daily doses. Palliative Medicine, 25(7), 725–732. http://doi.org/10.1177/0269216311398300

© 2019 Ladha KS et al. *JAMA Network Open.*

eTable 3. Baseline Characteristics for Patients by Surgical Procedure
3a. Baseline characteristics for patients undergoing laparoscopic cholecystectomies

	US		Canada		Sweden		P Value**
	n(%)	[95% CI]	n(%)	[95% CI]	n(%)	[95% CI]	
Total Number of Patients, n	46,781		39,098		3,530		
Sex (Male)	13,702 (29.3%)	(28.9-29.7)	9,856 (25.2%)	(24.8-25.6)	1,071 (30.3%)	(28.8-31.8)	<0.001
Age on date of procedure							
Mean (SD)	44.8 (12.1)	(44.7-44.9)	44.4 (12.2)	(44.2-44.5)	44.3 (12.1)		<0.001
18-39	16,631 (35.6%)	(35.1-36.0)	13,958 (35.7%)	(35.2-36.2)	1,255 (35.6%)	(34.0-37.2)	0.3679
40-54	18,653 (39.9%)	(39.4-40.3)	15,344 (39.2%)	(38.8-39.7)	1,393 (39.5%)	(37.9-41.1)	
55-64	11,497 (24.6%)	(24.2-25.0)	9,796 (25.1%)	(24.6-25.5)	882 (25%)	(23.6-26.4)	
Charlson Comorbidity Score*							
0	37,801 (80.8%)	(80.4-81.2)	36,993 (94.6%)	(94.4-94.8)	3,330 (94.3%)	(93.5-95.1)	<0.001
1	2,695 (5.8%)	(5.5-6.0)	1,730 (4.4%)	(4.2-4.6)	98 (2.8%)	(2.3-3.3)	
2 or more	6,285 (13.4%)	(13.1-13.7)	375 (1.0%)	(0.9-1.1)	102 (2.9%)	(2.3-3.5)	
Overnight hospital stay in 90 days before admission	1,894 (4.0%)	(3.9-4.2)	2,759 (7.1%)	(6.8-7.3)	349 (9.9%)	(8.9-10.9)	<0.001
Length of hospital stay associated with surgery (days), n (%)							
0	35,989 (76.9%)	(76.5-77.3)	30,195 (77.2%)	(76.8-77.6)	1,353 (38.3%)	(36.7-39.9)	<0.001
1	1,855 (4.0%)	(3.8-4.1)	2,982 (7.6%)	(7.4-7.9)	862 (24.4%)	(23.0-25.8)	
2 or more	8,937 (19.1%)	(18.7-19.5)	5,921 (15.1%)	(14.8-15.5)	1,315 (37.3%)	(35.7-38.9)	

*calculated based on data from the index surgical stay only
**Chi-squared test for categorical variables or ANOVA for continuous
SD = standard deviation; CI= confidence interval

© 2019 Ladha KS et al. *JAMA Network Open.*

3b. Baseline characteristics for patients undergoing laparoscopic appendectomies

	US		Canada		Sweden		P Value**
	n (%)	[95% CI]	n (%)	[95% CI]	n (%)	[95% CI]	
Total Number of Patients, n	22,368		20,437		2,234		
Sex (Male)	11,569 (51.7%)	(51.1-52.4)	10,352 (50.7%)	(50.0-51.3)	1,017 (45.5%)	(43.4-47.6)	<0.001
Age on date of procedure							
Mean (SD)	38.0 (13.0)	(37.8-38.1)	36.6 (13.2)	(36.4-36.8)	34.4 (12.7)		<0.001
18-39	12,986 (58.1%)	(57.4-58.7)	12,301 (60.2%)	(59.5-60.9)	1,521 (68.1%)	(66.2-70.0)	<0.001
40-54	6,441 (28.8%)	(28.2-29.4)	5,617 (27.5%)	(26.9-28.1)	489 (21.9%)	(20.2-23.6)	
55-64	2,941 (13.1%)	(12.7-13.6)	2,519 (12.3%)	(11.9-12.8)	224 (10%)	(8.8-11.2)	
Charlson Comorbidity Score*							
0	20,023 (89.5%)	(89.1-89.9)	19,640 (96.1%)	(95.8-96.4)	2,132 (95.4%)	(94.5-96.3)	<0.001
1	694 (3.1%)	(2.9-3.3)	571 (2.8%)	(2.6-3.0)	44 (2%)	(1.4-2.6)	
2 or more	1,651 (7.4%)	(7.0-7.7)	226 (1.1%)	(1.0-1.2)	58 (2.6%)	(1.9-3.3)	
Overnight hospital stay in 90 days before admission	346 (1.5%)	(1.4-1.7)	419 (2.1%)	(1.9-2.2)	72 (3.2%)	(2.5-3.9)	<0.001
Length of hospital stay associated with surgery (days), n (%)							
0	15,901 (71.1%)	(70.5-71.7)	2,181 (10.7%)	(10.2-11.1)	70 (3.1%)	(2.4-3.8)	<0.001
1	2,812 (12.6%)	(12.1-13.0)	10,981 (53.7%)	(53.0-54.4)	1,060 (47.4%)	(45.3-49.5)	
2 or more	3,655 (16.3%)	(15.9-16.8)	7275 (35.6%)	(34.9-36.3)	1,104 (49.4%)	(47.3-51.5)	

*calculated based on data from the index surgical stay only
**Chi-squared test for categorical variables or ANOVA for continuous
SD = standard deviation; CI= confidence interval

© 2019 Ladha KS et al. *JAMA Network Open.*

3c. Baseline characteristics for knee meniscectomies

	US		Canada		Sweden		P Value**
	n(%)	[95% CI]	n(%)	[95% CI]	n(%)	[95% CI]	
Total Number of Patients, n	44,060		6,975		1,791		
Sex (Male)	26,481 (60.1%)	(59.6-60.6)	4,565 (65.4%)	(64.3-66.6)	1,201 (67.1%)	(64.9-69.3)	<0.001
Age on date of procedure							
Mean (SD)	48.0 (12.1)	(47.9-48.1)	45.9 (11.9)	(45.6-46.2)	42.2 (12.2)		<0.001
18-39	10,062 (22.8%)	(22.4-23.2)	1,887 (27.1%)	(26.0-28.1)	695 (38.8%)	(36.5-41.1)	<0.001
40-54	19,044 (43.2%)	(42.8-43.7)	3,184 (45.6%)	(44.5-46.8)	789 (44.1%)	(41.8-46.4)	
55-64	14,954 (33.9%)	(33.5-34.4)	1,904 (27.3%)	(26.3-28.3)	307 (17.1%)	(15.4-18.8)	
Charlson Comorbidity Score*							
0	41,195 (93.5%)	(93.3-93.7)	6,775 (97.1%)	(96.7-97.5)	1,789 (99.9%)	(99.8-100.0)	0.003
1	1,258 (2.9%)	(2.7-3.0)	176 (2.5%)	(2.2-2.9)	NR		
2 or more	1,607 (3.6%)	(3.5-3.8)	24 (0.3%)	(0.2-0.5)	NR		
Overnight hospital stay in 90 days before admission	151 (0.3%)	(0.3-0.4)	10 (0.1%)	(0.1-0.2)	NR		<0.001
Length of hospital stay associated with surgery (days), n (%)							
0	43,963 (99.8%)	(99.7-99.8)	6,942 (99.5%)	(99.4-99.7)	1,759 (98.2%)	(97.6-98.8)	<0.001
1	23 (0.1%)	(0.0-0.1)	23 (0.3%)	(0.2-0.5)	21 (1.2%)	(0.7-1.7)	
2 or more	74 (0.2%)	(0.1-0.2)	10 (0.1%)	(0.1-0.2)	11 (0.6%)	(0.2-1.0)	

*calculated based on data from the index surgical stay only
**Chi-squared test for categorical variables or ANOVA for continuous
SD = standard deviation; CI= confidence interval; NR= not reportable

© 2019 Ladha KS et al. *JAMA Network Open.*

3d. Baseline characteristics for partial breast excisions

	US		Canada		Sweden		P Value**
	n(%)	[95% CI]	n(%)	[95% CI]	n(%)	[95% CI]	
Total Number of Patients, n	16,170		18,143		2,247		
Sex (Male)	675 (4.2%)	(3.9-4.5)	301 (1.7%)	(1.5-1.8)	25 (1.1%)	(0.7-1.5)	<0.001
Age on date of procedure							
Mean (SD)	47.5 (12.1)	(47.3-47.7)	48.5 (11.4)	(48.4-48.7)	48.0 (11.5)		<0.001
18-39	3,893 (24.1%)	(23.4-24.7)	3,482 (19.2%)	(18.6-19.8)	424 (18.9%)	(17.3-20.5)	<0.001
40-54	7,068 (43.7%)	(42.9-44.5)	8,326 (45.9%)	(45.2-46.6)	1,080 (48.1%)	(46.0-50.2)	
55-64	5,209 (32.2%)	(31.5-32.9)	6,335 (34.9%)	(34.2-35.6)	743 (33.1%)	(31.2-35.0)	
Charlson Comorbidity Score*							
0	9,549 (59.1%)	(58.3-59.8)	9,906 (54.6%)	(53.9-55.3)	1,136 (50.6%)	(48.5-52.7)	<0.001
1	285 (1.8%)	(1.6-2.0)	269 (1.5%)	(1.3-1.7)	NR	NR	
2 or more	6,336 (39.2%)	(38.4-39.9)	7,968 (43.9%)	(43.2-44.6)	NR	NR	
Overnight hospital stay in 90 days before admission	146 (0.9%)	(0.8-1.0)	91 (0.5%)	(0.4-0.6)	28 (1.2%)	(0.7-1.7)	<0.001
Length of hospital stay associated with surgery (days), n (%)							
0	16,030 (99.1%)	(99.0-99.3)	17638 (97.2%)	(97.0-97.5)	1,560 (69.4%)	(67.5-71.3)	<0.001
1	52 (0.3%)	(0.2-0.4)	435 (2.4%)	(2.2-2.6)	605 (26.9%)	(25.1-28.7)	
2 or more	88 (0.5%)	(0.4-0.7)	70 (0.4%)	(0.3-0.5)	82 (3.6%)	(2.8-4.4)	

*calculated based on data from the index surgical stay only
**Chi-squared test for categorical variables or ANOVA for continuous
NA = not applicable; SD = standard deviation; CI= confidence interval; NR= not reportable

© 2019 Ladha KS et al. *JAMA Network Open.*

eTable 4. First Postoperative Opioid Prescription Between Day 0 and Day 6 by Surgical Procedure
4a: First postoperative opioid prescription between day 0 and day 6 for all surgical procedures

	US<br>N=129,379		Canada<br>N=84,653		Sweden<br>N=9,802		P Value*
	n (%)	95% CI	n (%)	95% CI	n (%)	95% CI	
Opioid Prescription Filled	98,594 (76.2%)	(76.0-76.4)	66,544 (78.6%)	(78.3-78.9)	1,086 (11.1%)	(10.5-11.7)	<0.001
Among Those Who Filled an Opioid Prescription Within 7 Days							
	N=98,594		N=66,544		N=1,086		
Morphine Equivalents Dispensed (Milligrams), median (IQR)	225 (150-300)		135 (113-225)		105 (100-225)		NA
Morphine Equivalents Dispensed (Milligrams), mean (SD)	247 (145)		169 (93.1)		197 (191)		<0.001
Morphine Equivalents Dispensed (Milligrams)							
1-100	8,377 (8.5%)	(8.3-8.7)	11,487 (17.3%)	(17.0-17.6)	318 (29.3%)	(26.7-32.1)	<0.001
101-150	25,224 (25.6%)	(25.3-25.9)	28,798 (43.3%)	(42.9-43.7)	325 (29.9%)	(27.3-32.7)	
151-200	12,088 (12.3%)	(12.1-12.5)	6,813 (10.2%)	(10.0-10.5)	25 (2.3%)	(1.6-3.4)	
>200	52,905 (53.7%)	(53.3-54.0)	19,446 (29.2%)	(28.9-29.6)	418 (38.5%)	(35.6-41.4)	
Codeine	3,210 (3.3%)	(3.1-3.4)	26,136 (39.3%)	(38.9-39.6)	170 (15.7%)	(15.0-16.4)	<0.001
Hydromorphone	817 (0.8%)	(0.8-0.9)	5,819 (8.7%)	(8.5-9.0)	NA	NA	
Hydrocodone	54,956 (55.7%)	(55.4-56.0)	NA	NA	NA	NA	
Morphine	27 (0.0%)	(0.0-0.0)	1,025 (1.5%)	(1.4-1.6)	47 (4.3%)	(3.9-4.7)	
Oxycodone	34,378 (34.9%)	(34.6-35.2)	20,250 (30.4%)	(30.1-30.8)	513 (47.2%)	(46.2-48.2)	
Tramadol	3,425 (3.5%)	(3.4-3.6)	12,285 (18.5%)	(18.2-18.8)	315 (29.0%)	(28.1-29.9)	
Multiple	1,390 (1.4%)	(1.3-1.5)	877 (1.3%)	(1.2-1.4)	27 (2.5%)	(2.2-2.8)	
Other	391 (0.4%)	(0.4-0.4)	152 (0.2%)	(0.2-0.3)	14 (1.3%)	(1.1-1.5)	
Prescription Filled on Day 0	73,728 (74.8%)	(74.5-75.1)	61,421 (92.3%)	(92.1-92.5)	737 (67.9%)	(67.0-68.8)	<0.001
Combination Product Prescribed	89,390 (90.7%)	(90.5-90.8)	57,346 (86.2%)	(85.9-86.4)	171 (15.7%)	(15.0-16.4)	<0.001

* Chi-squared test for categorical variables or ANOVA for continuous
IQR = interquartile range; NA = not applicable; CI = confidence interval; SD = standard deviation

© 2019 Ladha KS et al. *JAMA Network Open.*

4b: First postoperative opioid prescription between day 0 and day 6 for laparoscopic cholecystectomy

	US N=46,781		Canada N=39,098		Sweden N=3,530		P Value*
	n(%)	95% CI	n(%)	95% CI	n(%)	95% CI	
Opioid Prescription Filled	35,447 (75.8%)	(75.4-76.2)	31,836 (81.4%)	(81.0-81.8)	559 (15.8%)	(14.7-17.1)	<0.001
Among Those Who Filled an Opioid Prescription Within 7 Days							
	N=35,447		N=31,836		N=559		
Morphine Equivalents Dispensed (Milligrams), median (IQR)	200 (150-263)		135 (113-225)		105 (100-225)		NA
Morphine Equivalents Dispensed (Milligrams), mean (SD)	220 (106)		164 (83.2)		204 (209)		<0.001
Morphine Equivalents Dispensed (Milligrams)							
1-100	2,933 (8.3%)	(8.0-8.6)	5,318 (16.7%)	(16.3-17.1)	155 (27.7%)	(24.2-31.6)	<0.001
101-150	10,603 (29.9%)	(29.4-30.4)	14,346 (45.1%)	(44.5-45.6)	178 (31.8%)	(28.1-35.8)	
151-200	4,205 (11.9%)	(11.5-12.2)	3,106 (9.8%)	(9.4-10.1)	11 (2.0%)	(1.1-3.5)	
>200	17,706 (50.0%)	(49.4-50.5)	9,066 (28.5%)	(28.0-29.0)	215 (38.5%)	(34.5-42.6)	
Type of Opioid Prescribed							
Codeine	1,132 (3.2%)	(3.0-3.4)	12,523 (39.3%)	(38.8-40.0)	37 (6.6%)	(5.8-7.4)	<0.001
Hydromorphone	402 (1.1%)	(1.0-1.2)	2,870 (9.02%)	(8.7-9.3)	NA	NA	
Hydrocodone	19,039 (53.7%)	(53.2-54.2)	NA	NA	NA	NA	
Morphine	15 (0.0%)	(0.0-0.1)	442 (1.4%)	(1.3-1.5)	31 (5.5%)	(4.7-6.3)	
Oxycodone	13,055 (36.8%)	(36.3-37.3)	9,723 (30.5%)	(30.0-31.0)	292 (52.2%)	(50.6-53.8)	
Tramadol	1,351 (3.8%)	(3.6-4.0)	6,035 (19.0%)	(18.5-19.4)	165 (29.5%)	(28.0-31.0)	
Multiple	306 (0.9%)	(0.8-1.0)	134 (0.4%)	(0.4-0.5)	22 (3.9%)	(3.3-4.5)	
Other	147 (0.4%)	(0.3-0.5)	109 (0.3%)	(0.3-0.4)	12 (2.1%)	(1.6-2.6)	
Prescription Filled on Day 0	25,366 (71.6%)	(71.1-72.0)	29,199 (91.7%)	(91.4-92.0)	408 (73%)	(71.5-74.5)	<0.001
Combination Product Prescribed	31,917 (90.0%)	(89.7-90.4)	27,344 (85.9%)	(85.5-86.2)	37 (6.6%)	(5.8-7.4)	<0.001

* Chi-squared test for categorical variables or ANOVA for continuous
IQR = interquartile range; NA = not applicable; CI = confidence interval; SD = standard deviation; NR= not reportable

© 2019 Ladha KS et al. *JAMA Network Open.*

4c: First postoperative opioid prescription between day 0 and day 6 for laparoscopic appendectomy

	US N=22,368		Canada N=20,437		Sweden N=2,234		P Value*
	n (%)	95% CI	n (%)	95% CI	n (%)	95% CI	
Opioid Prescription Filled	17,171 (76.8%)	(76.2-77.3)	15,515 (75.9%)	(75.3-76.5)	360 (16.1%)	(14.6-17.7)	<0.001
Among Those Who Filled an Opioid Prescription Within 7 Days							
	N=17,171		N=15,515		N=360		
Morphine Equivalents Dispensed (Milligrams), median (IQR)	200 (150-225)		135	(113-225)	105 (100-210)		NA
Morphine Equivalents Dispensed (Milligrams), mean (SD)	212 (104)	(210-214)	158 (77.8)	(157-160)	178 (180)		<0.001
Morphine Equivalents Dispensed (Milligrams)							
1-100	1,709 (10.0%)	(9.5-10.4)	3,066 (19.8%)	(19.1-20.4)	105 (29.2%)	(24.7-34.1)	<0.001
101-150	5,416 (31.5%)	(30.9-32.2)	7,074 (45.6%)	(44.8-46.4)	NR	NR	
151-200	1,882 (11.0%)	(10.5-11.4)	1,185 (7.6%)	(7.2-8.1)	NR	NR	
>200	8,164 (47.5%)	(46.8-48.3)	4,190 (27.0%)	(26.3-27.7)	113 (31.4%)	(26.8-36.4)	
Type of Opioid Prescribed							
Codeine	562 (3.3%)	(3.0-3.5)	5,130 (33.1%)	(32.3-33.8)	50 (13.9%)	(12.5-15.3)	<0.001
Hydromorphone	141 (0.8%)	(0.7-1.0)	1,786 (11.5%)	(11.0-12.0)	NA	NA	
Hydrocodone	9,115 (53.1%)	(52.3-53.8)	NA	NA	NA	NA	
Morphine	2 (0.0%)	(0.0-0.0)	442 (2.8%)	(2.6-3.1)	10 (2.8%)	(2.1-3.5)	
Oxycodone	6,634 (38.6%)	(37.9-39.4)	5,295 (34.1%)	(33.4-34.9)	189 (52.5%)	(50.4-54.6)	
Tramadol	562 (3.3%)	(3.0-3.5)	2,742 (17.7%)	(17.1-18.3)	106 (29.4%)	(27.5-31.3)	
Multiple	126 (0.7%)	(0.6-0.9)	114 (0.7%)	(0.6-0.9)	NR	NR	
Other	29 (0.2%)	(0.1-0.2)	6 (0.04%)	(0.0-0.1)	NR	NR	
Prescription Filled on Day 0	6,893 (40.1%)	(39.4-40.9)	14,246 (91.8%)	(91.4-92.3)	302 (83.9%)	(82.4-85.4)	<0.001
Combination Product Prescribed	15,475 (90.1%)	(89.7-90.6)	12,582 (81.1%)	(80.5-81.7)	51 (14.2%)	(12.8-15.6)	<0.001

* Chi-squared test for categorical variables or ANOVA for continuous
IQR = interquartile range; NA = not applicable; CI = confidence interval; SD = standard deviation; NR= not reportable

© 2019 Ladha KS et al. *JAMA Network Open.*

4d: First postoperative opioid prescription between day 0 and day 6 for knee meniscectomy

	US N=44,060		Canada N=6,975		Sweden N=1,791		P Value*
	n (%)	95% CI	n (%)	95% CI	n (%)	95% CI	
Opioid Prescription Filled	35,006 (79.5%)	(79.1-79.8)	5,590 (80.1%)	(79.2-81.1)	121 (6.8%)	(5.7-8.0)	<0.001
Among Those Who Filled an Opioid Prescription Within 7 Days							
	N=35,006		N=5,590		N=121		
Morphine Equivalents Dispensed (Milligrams), median (IQR)	300 (200-400)		225	(135- 315)	210 (100-225)		NA
Morphine Equivalents Dispensed (Milligrams), mean (SD)	314 (179)	(312-316)	261 (154)	(257-265)	218 (146)		<0.001
Morphine Equivalents Dispensed (Milligrams)							
1-100	1,117 (3.2%)	(3.0-3.4)	447 (8.0%)	(7.3-8.7)	44 (36.4%)	(28.3-45.2)	<0.001
101-150	5,189 (14.8%)	(14.5-15.2)	1,256 (22.5%)	(21.4-23.6)	NR		
151-200	4,918 (14.0%)	(13.7-14.4)	874 (15.6%)	(14.7-16.6)	NR		
>200	23,782 (67.9%)	(67.4-68.4)	3,013 (53.9%)	(52.6-55.2)	66 (54.5%)	(45.7-63.1)	
Type of Opioid Prescribed							
Codeine	911 (2.6%)	(2.4-2.8)	2,359 (42.2%)	(40.9-43.5)	75 (62%)	(59.8-64.2)	<0.001
Hydromorphone	178 (0.5%)	(0.4-0.6)	322 (5.8%)	(5.1-6.4)	NA	NA	
Hydrocodone	20,422 (58.3%)	(57.8-58.9)	NA	NA	NA	NA	
Morphine	8 (0.0%)	(0.0-0.0)	41 (0.7%)	(0.5-1.0)	NR	NR	
Oxycodone	11,556 (33.0%)	(32.5-33.5)	1,617 (28.9%)	(27.7-30.1)	16 (13.2%)	(11.6-14.8)	
Tramadol	850 (2.4%)	(2.3-2.6)	655 (11.7%)	(10.9-12.6)	28 (23.1%)	(21.1-25.1)	
Multiple	899 (2.6%)	(2.4-2.7)	588 (10.5%)	(9.7-11.3)	NR	NR	
Other	182 (0.5%)	(0.4-0.6)	8 (0.1%)	(0.0-0.2)	0	-	
Prescription Filled on Day 0	32,274 (92.2%)	(91.9-92.5)	5,232 (93.6%)	(93.0-94.2)	13 (10.7%)	(9.3-12.1)	<0.001
Combination Product Prescribed	32,080 (91.6%)	(91.4-91.9)	5,169 (92.5%)	(91.8-93.2)	75 (62%)	(59.8-64.2)	<0.001

* Chi-squared test for categorical variables or ANOVA for continuous
IQR = interquartile range; NA = not applicable; CI = confidence interval; SD = standard deviation; NR= not reportable

© 2019 Ladha KS et al. *JAMA Network Open.*

4e: First postoperative opioid prescription between day 0 and day 6 for partial breast excision

	US N=16,170		Canada N=18,143		Sweden N=2,247		P Value*
	n (%)	95% CI	n (%)	95% CI	n (%)	95% CI	
Opioid Prescription Filled	10,970 (67.8%)	(67.1-68.6)	13,603 (75.0%)	(74.3-75.6)	46 (2.0%)	(1.5-2.7)	<0.001
Among Those Who Filled an Opioid Prescription Within 7 Days							
	N=10,970		N=13,603		N=46		
Morphine Equivalents Dispensed (Milligrams), median (IQR)	150 (113-225)		135	(113-188)	210 (100-250)		NA
Morphine Equivalents Dispensed (Milligrams), mean (SD)	175 (93.7)	(173-177)	155 (75)	(154-156)	216 (144)		<0.001
Morphine Equivalents Dispensed (Milligrams)							
1-100	2,618 (23.9%)	(23.1-24.7)	2,656 (19.5%)	(18.9-20.2)	14 (30.4%)	(19.1-44.8)	<0.001
101-150	4,016 (36.6%)	(35.7-37.5)	6,122 (45.0%)	(44.2-45.8)	8 (17.4%)	(9.1-30.7)	
151-200	1,083 (9.9%)	(9.3-10.4)	1,648 (12.1%)	(11.6-12.7)	0	NA	
>200	3,253 (29.7%)	(28.8-30.5)	3,177 (23.4%)	(22.7-24.1)	24 (52.2%)	(38.1-65.9)	
Type of Opioid Prescribed							
Codeine	605 (5.5%)	(5.1-5.9)	6,124 (45.0%)	(44.2-45.9)	8 (17.4%)	(15.8-19.0)	<0.001
Hydromorphone	96 (0.9%)	(0.7-1.0)	841 (6.2%)	(5.8-6.6)	NA	NA	
Hydrocodone	6,380 (58.2%)	(57.2-59.1)	NA	NA	NA	NA	
Morphine	2 (0.0%)	(0.0-0.0)	100 (0.7%)	(0.6-0.9)	NR	NR	
Oxycodone	3,133 (28.6%)	(27.7-29.4)	3,615 (26.6%)	(25.8-27.3)	16 (34.8%)	(32.8-36.8)	
Tramadol	662 (6.0%)	(5.6-6.5)	2,853 (21.0%)	(20.3-21.7)	16 (34.8%)	(32.8-36.8)	

© 2019 Ladha KS et al. *JAMA Network Open.*

Multiple	59 (0.5%)	(0.4-0.7)	41 (0.3%)	(0.2-0.4)	0	-	
Other	33 (0.3%)	(0.2-0.4)	29 (0.2%)	(0.1-0.3)	NR	NR	
Prescription Filled on Day 0	9,195 (83.8%)	(83.1-84.5)	12,744 (93.7%)	(93.3-94.1)	14 (30.4%)	(28.5-32.3)	<0.001
Combination Product Prescribed	9,918 (90.4%)	(89.9-91.0)	12,251 (90.1%)	(89.6-90.6)	NR	NR	<0.001

* Chi-squared test for categorical variables or ANOVA for continuous
IQR = interquartile range; NA = not applicable; CI = confidence interval; SD = standard deviation; NA = not applicable; NR= not reportable

© 2019 Ladha KS et al. *JAMA Network Open.*

eTable 5. Morphine Milligram Equivalents Prescribed in First Postoperative Opioid Prescription Between Day 0 and Day 6 by Surgical Procedure

5a. Morphine milligram equivalents prescribed in first postoperative opioid prescription between day 0 and day 6 for all surgical procedures

	US n=98,594		Canada n=66,544		Sweden n=1,086		P Value*
Type of Opioid Prescribed	Mean (SD)	Median (IQR)	Mean (SD)	Median (IQR)	Mean (SD)	Median (IQR)	
Codeine	147 (68.2)	135 (108-180)	138 (59.9)	135 (113-135)	197 (191)	225 (90-225)	<0.001
Hydromorphone	302 (156)	240 (240-320)	141 (97.3)	120 (80-160)	NA	NA	<0.001
Hydrocodone	215 (115)	200 (150-250)	NA	NA	NA	NA	
Morphine	444 (269)	450 (225-600)	149 (72.4)	150 (100-150)	323 (173)	250 (250-300)	<0.001
Oxycodone	301 (147)	263 (225-338)	236 (80.9)	225 (225-300)	211 (233)	105 (105-210)	<0.001
Tramadol	160 (72.1)	150 (113-200)	117 (42)	113 (113-113)	151 (108)	100 (100-200)	<0.001
Multiple	534 (299)	450 (350-675)	473 (181)	570 (330-570)	307 (279)	205 (101-460)	<0.001
Other	456 (381)	278 (150-600)	210 (149)	150 (100-300)	100 (0)	100 (100-100)	<0.001

* ANOVA

IQR = interquartile range; SD = standard deviation; NA = not applicable; NR= not reportable

© 2019 Ladha KS et al. *JAMA Network Open.*

5b. Morphine milligram equivalents prescribed in first postoperative opioid prescription between day 0 and day 6 for laparoscopic cholecystectomy

	US n=35,447		Canada n=31,836		Sweden n=559		P Value*
Type of Opioid Prescribed	Mean (SD)	Median (IQR)	Mean (SD)	Median (IQR)	Mean (SD)	Median (IQR)	
Codeine	144 (61.0)	135 (113-180)	138 (62.8)	135 (113-135)	204 (209)	225 (90-225)	<0.001
Hydromorphone	287 (145)	240 (240-320)	143 (106)	120 (80-160)	NA	NA	<0.001
Hydrocodone	192 (84.3)	150 (150-225)	NA	NA	NA	NA	
Morphine	414 (233)	450 (225-450)	158 (74.2)	150 (100-200)	348 (202)	250 (250-500)	<0.001
Oxycodone	267 (109)	225 (225-300)	232 (70.3)	225 (225-300)	221 (247)	105 (105-210)	<0.001
Tramadol	157 (63.0)	150 (120-175)	115 (37.8)	113 (113-113)	139 (81)	100 (100-100)	<0.001
Multiple	367 (213)	333 (250-445)	315 (200)	275 (195-360)	321 (296)	206 (101-460)	0.053
Other	311 (259)	150 (150-555)	176 (128)	100 (100-150)	100 (0)	100 (100-100)	<0.001

* ANOVA

IQR = interquartile range; SD = standard deviation; NA = not applicable; NR= not reportable

© 2019 Ladha KS et al. *JAMA Network Open.*

5c. Morphine milligram equivalents prescribed in first postoperative opioid prescription between day 0 and day 6 for laparoscopic appendectomy

	US n=17,171		Canada n=15,515		Sweden n=360		P Value*
Type of Opioid Prescribed	Mean (SD)	Median (IQR)	Mean (SD)	Median (IQR)	Mean (SD)	Median (IQR)	
Codeine	142 (60.6)	135 (108-158)	130 (52.8)	135 (90-135)	178 (180)	146 (90-225)	<0.001
Hydromorphone	267 (130)	240 (192-320)	131 (82.8)	120 (80-160)	NA	NA	<0.001
Hydrocodone	184 (83.4)	150 (150-225)	NA	NA	NA	NA	
Morphine	338 (159)	338 (225-450)	136 (71.6)	150 (100-150)	288 (84)	250 (250-250)	<0.001
Oxycodone	255 (106)	225 (225-300)	218 (70.7)	225 (150-225)	190 (222)	105 (105-210)	<0.001
Tramadol	160 (71.9)	150 (113-200)	113 (44.6)	113 (90-113)	149 (119)	100 (100-100)	<0.001
Multiple	404 (273)	375 (285-450)	274 (117)	248 (195-360)	290 (196)	253 (148-433)	<0.001
Other	401 (249)	480 (150-600)	308 (177)	275 (150-500)	100 (0)	100 (100-100)	0.357

* ANOVA

IQR = interquartile range; SD = standard deviation; NA = not applicable; NR= not reportable

© 2019 Ladha KS et al. *JAMA Network Open.*

5d. Morphine milligram equivalents prescribed in first postoperative opioid prescription between day 0 and day 6 for knee meniscectomy

	US n=35,006		Canada n=6,975		Sweden n=121		P Value*
Type of Opioid Prescribed	Mean (SD)	Median (IQR)	Mean (SD)	Median (IQR)	Mean (SD)	Median (IQR)	
Codeine	176 (79.1)	135 (135-180)	174 (65.1)	162 (135-225)	218 (146)	225 (90-225)	<0.001
Hydromorphone	398 (180)	360 (320-480)	203 (115)	160 (120-288)	NA	NA	<0.001
Hydrocodone	269 (140)	225 (175-300)	NA	NA	NA	NA	
Morphine	497 (379)	443 (165-675)	155 (31.4)	150 (150-150)	NR	NR	<0.001
Oxycodone	385 (176)	338 (300-450)	336 (118)	300 (225-450)	256 (115)	210 (210-315)	<0.001
Tramadol	187 (79.8)	150 (150-210)	157 (56.3)	150 (113-187.5)	186 (137)	100 (100-225)	<0.001
Multiple	620 (300)	525 (390-750)	557 (109)	570 (570-570)	NR	NR	<0.001
Other	621 (437)	555 (200-900)	241 (130)	225 (138-300)	NA	NA	0.015

* ANOVA
IQR = interquartile range; SD = standard deviation; NR= not reportable

© 2019 Ladha KS et al. *JAMA Network Open.*

5e: Morphine milligram equivalents prescribed in first postoperative opioid prescription between day 0 and day 6 for partial breast excision

	US n=10,970		Canada n=13,603		Sweden n=46		P Value*
Type of Opioid Prescribed	Mean (SD)	Median (IQR)	Mean (SD)	Median (IQR)	Mean (SD)	Median (IQR)	
Codeine	115 (50.9)	113 (90.0-135)	131 (52.0)	135 (90-135)	216 (144)	225 (90-225)	<0.001
Hydromorphone	242 (103)	240 (160-320)	133 (78.8)	120 (80-160)	NA	NA	<0.001
Hydrocodone	153 (69.7)	150 (100-175)	NA	NA	NA	NA	
Morphine	563 (53.0)	563 (525-600)	158 (72.5)	150 (113-200)	255 (76)	300 (250-300)	<0.001
Oxycodone	235 (108)	225 (169-300)	228 (68.9)	225 (188-263)	230 (159)	210 (105-315)	0.006
Tramadol	131 (66.8)	125 (100-150)	117 (39.9)	113 (113-113)	219 (175)	100 (100-375)	<0.001
Multiple	363 (159)	350 (275-400)	345 (228)	315 (255-360)	NA	NA	0.643
Other	242 (177)	150 (125-300)	312 (170)	300 (185-463)	100 (0)	100 (100-100)	0.183

* ANOVA
IQR = interquartile range; SD = standard deviation; NR= not reportable

© 2019 Ladha KS et al. *JAMA Network Open.*

eTable 6. Opioid Prescriptions Filled Within 30 Days of Surgery or Date of Hospital Discharge by Surgical Procedure

6a. Opioid prescriptions filled within 30 days of surgery or date of hospital discharge for all procedures

	**US N=129,379**		**Canada N=84,653**		**Sweden N=9,802**		**P Value***
	n (%)	95% CI	n (%)	95% CI	n (%)	95% CI	
Morphine Equivalents Dispensed (Milligrams)							
0	27,844 (21.5%)	(21.3-21.7)	17,487 (20.7%)	(20.4-20.9)	8,591 (87.6%)	(86.9-88.3)	<0.001
1-100	7,735 (6.0%)	(5.8-6.1)	10,910 (12.9%)	(12.7-13.1)	329 (3.4%)	(3.0-3.8)	
101-150	23,336 (18.0%)	(17.8-18.2)	27,535 (32.5%)	(32.2-32.8)	321 (3.3%)	(2.9-3.7)	
151-200	11,597 (9.0%)	(8.8-9.1)	6,790 (8.0%)	(7.8-8.2)	35 (0.4%)	(0.3-0.5)	
>200	58,867 (45.5%)	(45.2-45.8)	21,931 (25.9%)	(25.6-26.2)	526 (5.4%)	(5.0-5.8)	
Among Those Who Filled an Opioid Prescription within 30-days							
	N=101,535		N=67,166		N=1,211		
Morphine Equivalents Dispensed (Milligrams), median (IQR)*	225 (150-300)		150 (113-225)		105 (100-225)		NA
Morphine Equivalents Dispensed (Milligrams), mean (SD)*	283 (217)		183 (140)		229 (295)		<0.001
2 or More Prescriptions Filled	13,291 (13.1%)	(12.9-13.3)	4,828 (7.2%)	(7.0-7.4)	157 (13.0%)	(12.3-13.7)	<0.001

* Chi-squared test for categorical variables or ANOVA for continuous

IQR = interquartile range; NA = not applicable; CI = confidence interval; SD = standard deviation

© 2019 Ladha KS et al. *JAMA Network Open.*

6b: Opioid prescriptions filled within 30 days of surgery or date of hospital discharge for laparoscopic cholecystectomy

	US N=46,781		Canada N=39,098		Sweden N=3,530		P Value*
	n (%)	95% CI	n (%)	95% CI	n (%)	95% CI	
Morphine Equivalents Dispensed (Milligrams)							
0	10,113 (21.6%)	(21.2-22.0)	7,029 (18.0%)	(17.6-18.4)	2,921 (82.7%)	(81.5-83.9)	<0.001
1-100	2,691 (5.8%)	(5.5-6.0)	5,033 (12.9%)	(12.5-13.2)	159 (4.5%)	(3.8-5.2)	
101-150	9,853 (21.1%)	(20.7-21.4)	13,756 (35.2%)	(34.7-35.7)	180 (5.1%)	(4.4-5.8)	
151-200	4,115 (8.8%)	(8.5-9.1)	3,108 (7.95%)	(7.7-8.2)	16 (0.5%)	(0.3-0.7)	
>200	20,009 (42.8%)	(42.3-43.2)	10,172 (26.0%)	(25.6-26.5)	254 (7.2%)	(6.3-8.1)	
Among Those Who Filled an Opioid Prescription within 30-days							
	N=36,668		N=32,069		N=609		
Morphine Equivalents Dispensed (Milligrams), median (IQR)	225 (150-300)		135 (113-225)		105 (100-250)		NA
Morphine Equivalents Dispensed (Milligrams), mean (SD)	247 (169)		177 (146)		247 (364)		<0.001
2 or More Prescriptions Filled	4,026 (11.0%)	(10.7-11.3)	1,881 (5.9%)	(5.6-6.1)	93 (15.3%)	(14.1-16.5)	<0.001

* Chi-squared test for categorical variables or ANOVA for continuous
IQR = interquartile range; NA = not applicable; CI = confidence interval; SD = standard deviation

© 2019 Ladha KS et al. *JAMA Network Open.*

6c: Opioid prescriptions filled within 30 days of surgery or date of hospital discharge for laparoscopic appendectomy

	US N=22,368		Canada N=20437		Sweden N=2,234		P Value*
	n (%)	95% CI	n (%)	95% CI	n (%)	95% CI	
Morphine Equivalents Dispensed (Milligrams)							
0	4,980 (22.3%)	(21.7-22.8)	4,786 (23.4%)	(22.8-24.0)	1,860 (83.3%)	(81.8-84.8)	<0.001
1-100	1,546 (6.9%)	(6.6-7.2)	2,899 (14.2%)	(13.7-14.7)	102 (4.6%)	(3.7-5.5)	
101-150	4,961 (22.2%)	(21.6-22.7)	6,729 (32.9%)	(32.3-33.6)	NR	NR	
151-200	1,835 (8.2%)	(7.8-8.6)	1,207 (5.9%)	(5.6-6.2)	NR	NR	
>200	9,046 (40.4%)	(39.8-41.1)	4,816 (23.6%)	(23.0-24.1)	134 (6%)	(5.0-7.0)	
Among Those Who Filled an Opioid Prescription within 30-days							
	N=17,388		N=15,561		N=374		
Morphine Equivalents Dispensed (Milligrams), median (IQR)	225 (150-300)		135 (113–225)		105 (100-225)		NA
Morphine Equivalents Dispensed (Milligrams), mean (SD)	236 (149)		172 (109)		192 (195)		<0.001
2 or More Prescriptions Filled	1,833 (10.5%)	(10.1-11.0)	1,144 (7.3%)	(6.9-7.7)	37 (9.9%)	(8.7-11.1)	<0.001

* Chi-squared test for categorical variables or ANOVA for continuous

IQR = interquartile range; NA = not applicable; CI = confidence interval; SD = standard deviation; NR= not reportable

© 2019 Ladha KS et al. *JAMA Network Open.*

6d: Opioid prescriptions filled within 30 days of surgery or date of hospital discharge for knee meniscectomy

	**US N=44,060**		**Canada N=6975**		**Sweden N=1,791**		**P Value***
	n (%)	95% CI	n (%)	95% CI	n (%)	95% CI	
Morphine Equivalents Dispensed (Milligrams)							
0	8,128 (18.4%)	(18.1-18.8)	1,312 (18.8%)	(17.9-19.7)	1,642 (91.7%)	(90.4-93.0)	<0.001
1-100	1,037 (2.4%)	(2.2-2.5)	433 (6.2%)	(5.6-6.8)	50 (2.8%)	(2.0-3.6)	
101-150	4,742 (10.8%)	(10.5-11.1)	1,219 (17.5%)	(16.6-18.4)	NR	NR	
151-200	4,520 (10.3%)	(10.0-10.5)	845 (12.1%)	(11.3-12.9)	NR	NR	
>200	25,633 (58.2%)	(57.7-58.6)	3,166 (45.4%)	(44.2-46.6)	86 (4.8%)	(3.8-5.8)	
Among Those Who Filled an Opioid Prescription within 30-days							
	N=35,932		N=5,663		N=149		
Morphine Equivalents Dispensed (Milligrams), median (IQR)	300 (200-450)		225 (135- 375)		210 (100-250)		NA
Morphine Equivalents Dispensed (Milligrams), mean (SD)	368 (270)		277 (183)		236 (173)		<0.001
2 or More Prescriptions Filled	6,003 (16.7%)	(16.3-17.1)	857 (15.1%)	(14.2-16.1)	13 (8.7%)	(7.4-10.0)	<0.001

* Chi-squared test for categorical variables or ANOVA for continuous

IQR = interquartile range; NA = not applicable; CI = confidence interval; SD = standard deviation; NR= not reportable

© 2019 Ladha KS et al. *JAMA Network Open.*

6e: Opioid prescriptions filled within 30 days of surgery or date of hospital discharge for partial breast excision

	**US N=16,170**		**Canada N=18,143**		**Sweden N=2,247**		**P Value***
	n (%)	95% CI	n (%)	95% CI	n (%)	95% CI	
Morphine Equivalents Dispensed (Milligrams)							
0	4,623 (28.6%)	(27.9-29.3)	4,360 (24.0%)	(23.4-24.7)	2,168 (96.5%)	(95.7-97.3)	<0.001
1-100	2,461 (15.2%)	(14.7-15.8)	2,545 (14.0%)	(13.5-14.5)	18 (0.8%)	(0.4-1.2)	
101-150	3,780 (23.4%)	(22.7-24.0)	5,831 (32.1%)	(31.5-32.8)	NR	NR	
151-200	1,127 (7.0%)	(6.6-7.4)	1,630 (9.0%)	(8.6-9.4)	NR	NR	
>200	4,179 (25.8%)	(25.2-26.5)	3,777 (20.8%)	(20.2-21.4)	52 (2.3%)	(1.7-2.9)	
Among Those Who Filled an Opioid Prescription within 30-days							
	N=11,547		N=13,783		N=79		
Morphine Equivalents Dispensed (Milligrams), median (IQR)	150 (125-225)		135 (113-225)		225 (105-350)		NA
Morphine Equivalents Dispensed (Milligrams), mean (SD)	206 (161)		172 (118)		284 (239)		<0.001
2 or More Prescriptions Filled	1,429 (12.4%)	(11.8-13.0)	946 (6.9%)	(6.5-7.3)	14 (17.7%)	(16.1-19.3)	<0.001

* Chi-squared test for categorical variables or ANOVA for continuous
IQR = interquartile range; NA = not applicable; CI = confidence interval; SD = standard deviation; NR= not reportable

© 2019 Ladha KS et al. *JAMA Network Open.*

eTable 7. Sensitivity Analysis Excluding Patients Hospitalized for Surgery (Outpatient Surgery Only)

	n	Opioid Prescription filled days 0-6 after discharge, n (%)	95% CI	P Value*
All procedures				
US	111,883	86,085 (76.9%)	(76.7-77.2)	<0.001
Canada	56,956	45,953 (80.7%)	(80.4-81.0)	
Sweden	4,742	223 (4.7%)	(4.1-5.3)	
Laparoscopic Cholecystectomy				
US	35,989	27,836 (77.3%)	(76.9-77.8)	<0.001
Canada	30,195	25,283 (83.7%)	(83.3-84.1)	
Sweden	1,353	84 (6.2%)	(4.9-7.5)	
Laparoscopic Appendectomy				
US	15,901	12,443 (78.3%)	(77.6-78.9)	<0.001
Canada	2,181	1,825 (83.7%)	(82.1-85.2)	
Sweden	70	NR		
Knee Meniscectomy				
US	43,963	34,938 (79.5%)	(79.1-79.8)	<0.001
Canada	6,942	5,567 (80.2%)	(79.3-81.1)	
Sweden	1,759	106 (6%)	(4.9-7.1)	
Breast Excision				
US	16,030	10,868 (67.8%)	(67.1-68.5)	<0.001
Canada	17,638	13,278 (75.3%)	(74.6-75.9)	
Sweden	1,560	25 (1.6%)	(1.0-2.2)	

NR= not reportable
*Chi-squared test

© 2019 Ladha KS et al. *JAMA Network Open.*

eFigure. **Distribution of Morphine Milligram Equivalents Dispensed for All Prescriptions Filled, Days 0 to 29, for Patients Who Filled an Opioid Prescription in Each Country, by Surgical Procedure**
Bars represent 95% confidence intervals

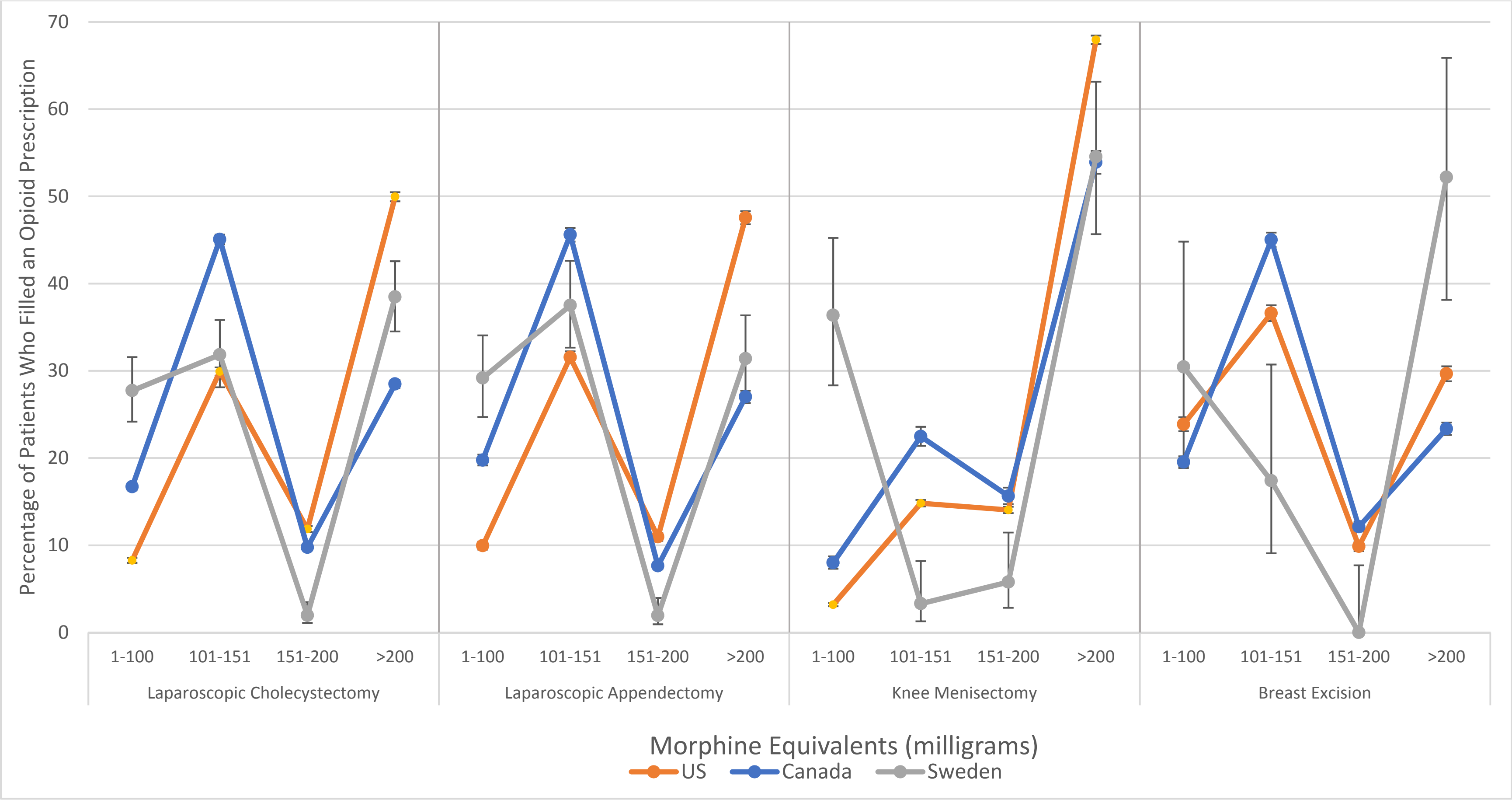

© 2019 Ladha KS et al. *JAMA Network Open.*
